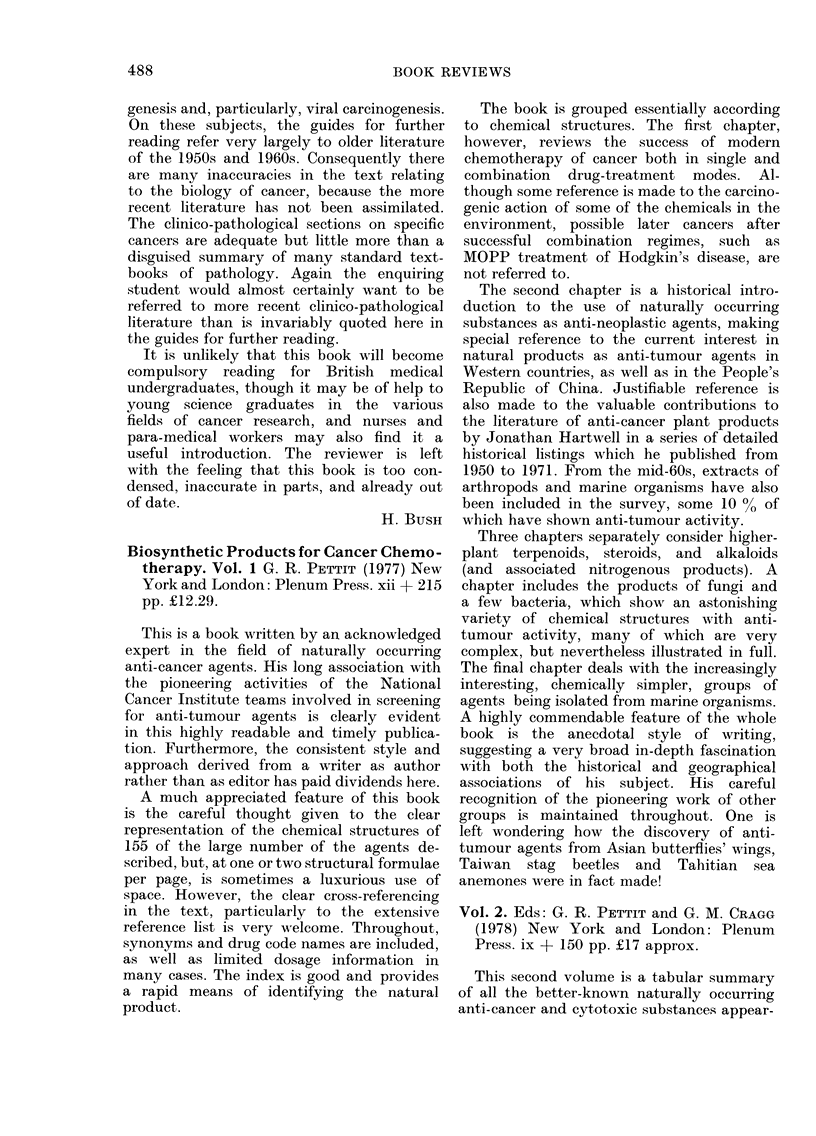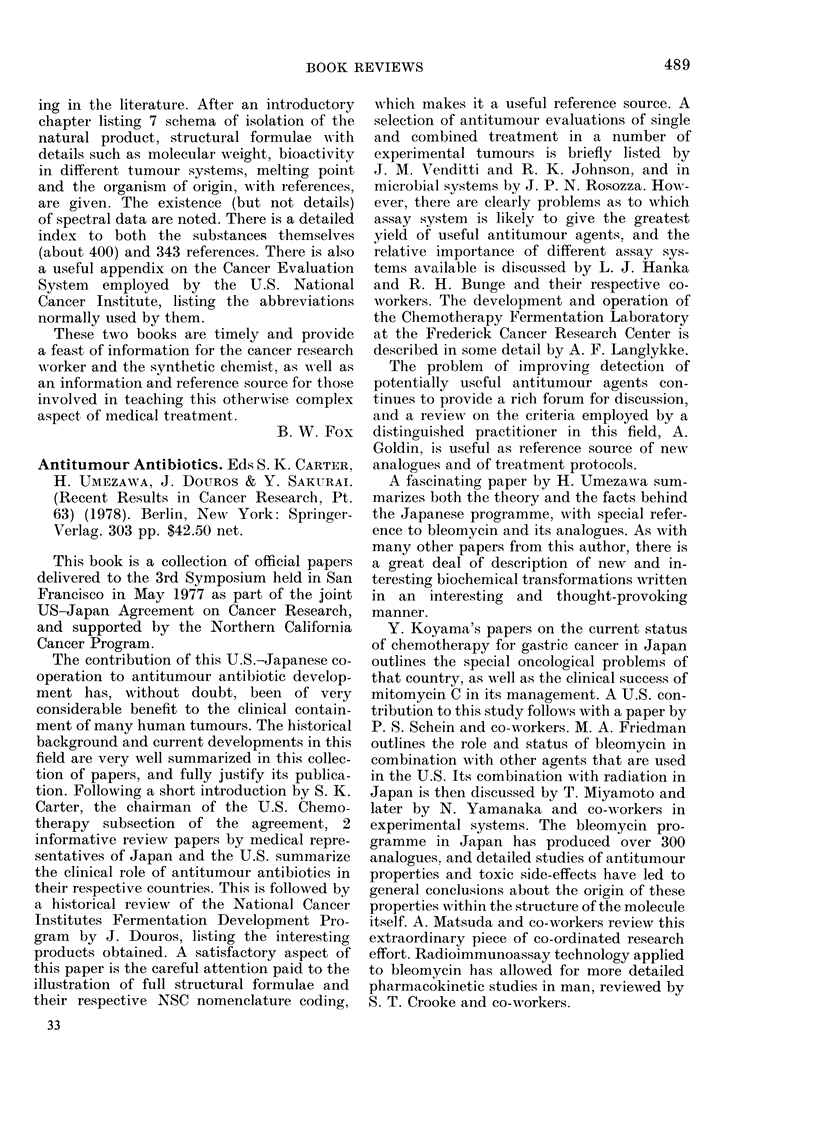# Biosynthetic Products for Cancer Chemotherapy. Vol. 2

**Published:** 1979-04

**Authors:** B. W. Fox


**Vol. 2.** Eds: G. R. PETTIT and G. M. CRAGG (1978) New York and London: Plenum Press. ix + 150 pp. £17 approx.

This second volume is a tabular summary of all the better-known naturally occurring anti-cancer and cytotoxic substances appearing in the literature. After an introductory chapter listing 7 schema of isolation of the natural product, structural formulae with details such as molecular weight, bioactivity in different tumour systems, melting point and the organism of origin, with references, are given. The existence (but not details) of spectral data are noted. There is a detailed index to both the substances themselves (about 400) and 343 references. There is also a useful appendix on the Cancer Evaluation System employed by the U.S. National Cancer Institute, listing the abbreviations normally used by them.

These two books are timely and provide a feast of information for the cancer research worker and the synthetic chemist, as well as an information and reference source for those involved in teaching this otherwise complex aspect of medical treatment.

B. W. Fox